# UHPLC Analysis of *Reynoutria japonica* Houtt. Rhizome Preparations Regarding Stilbene and Anthranoid Composition and Their Antimycobacterial Activity Evaluation

**DOI:** 10.3390/plants10091809

**Published:** 2021-08-30

**Authors:** Fabian Alperth, Lena Melinz, Johannes-Paul Fladerer, Franz Bucar

**Affiliations:** Institute of Pharmaceutical Sciences, University of Graz, Beethovenstraße 8, 8010 Graz, Austria; fabian.alperth@uni-graz.at (F.A.); lena.melinz@gmail.com (L.M.); johannes.fladerer@uni-graz.at (J.-P.F.)

**Keywords:** *Reynoutria japonica*, anthranoids, stilbenes, UHPLC-MS, UHPLC-DAD, MIC, antimicrobial, antimycobacterial, *Mycobacterium smegmatis*

## Abstract

*Reynoutria japonica* Houtt. is a critical invasive alien plant in Europe and North America with a drastic impact on native flora. However, *R. japonica* has medicinal potential, especially as a source of stilbenes. In order to explore the potential of simple extractions of *R. japonica*, we conducted qualitative and quantitative analyses of fresh *R. japonica* rhizome infusion, decoction, and macerates with ethanol by UHPLC-DAD-ESI-MS^n^ and UHPLC-DAD, with a focus on major constituent groups of stilbenes and anthranoids. Since *R. japonica* rhizome extracts showed antimicrobial potential in the past, we also evaluated the antimycobacterial effect of raw *R. japonica* extracts for the first time against *Mycobacterium smegmatis*. Of thirty-four characterized substances, six were stilbenes and twelve anthranoids. The main constituents, four trans-stilbenes and eight anthranoids, were quantified in a validated UHPLC-DAD method. The 38% ethanol macerate showed high stilbene (155.078 mg/100 g fluid extract) and low anthranoid content (5.420 mg/100 g fluid extract), while decoction showed the highest anthranoids. Antimycobacterial testing gave good results for all macerates (MIC 256 µg/mL) and *trans*-resveratrol (64 µg/mL). Extraction and enrichment of stilbenes from fresh plant material by simple extraction methods with food-grade solvents might encourage consideration of wild harvest of rhizomes over classic means of eradication of *R. japonica*.

## 1. Introduction

*Reynoutria japonica* Houtt. (Syn. *Fallopia japonica* (Houtt.) Ronse Decr., *Polygonum cuspidatum* Siebold and Zucc.) is an herbal perennial plant in the family of Polygonaceae [1]. The plant is native to eastern Asian regions of China, Japan, and Korea, but was introduced to the west as a garden plant in the 19th century [2]. It grows to heights of up to three metres in various conditions, and possesses thickened rhizomes, which may extend 4.5 m deep and up to 20 m away from parent plants [3,4]. Rhizomes regenerate easily even from small fragments, and therefore asexual dispersal forms the main way of reproduction in Europe and North America, often following natural disturbances or disposal of rhizome contaminated soil. Due to *R. japonica* being a fast-growing competitor early in the season, capturing space and resources, it is an invasive alien plant which can significantly diminish native flora [3,4]. However, in its native regions of China, *R. japonica* is also grown and harvested for long-lasting medicinal use in traditional Chinese medicine (TCM) [5]. Dried rhizome is called “Hu Zhang” in Chinese and listed in the Pharmacopeia of the People’s Republic of China, as well as the European Pharmacopeia [6,7]. Indications in TCM comprise jaundice, hepatitis, amenorrhoea and cough, among others [6]. In Korean folk medicine, *R. japonica* rhizome is used to support dental hygiene. Antibacterial effects against *Streptococcus mutans* and *Streptococcus sanguinis* are reported and considered to prevent dental plaque formation and therefore tooth decay, as well as accelerate wound healing on gums [8]. Major secondary metabolites in the constituent profile of *R. japonica* rhizomes are stilbenes, such as polydatin and resveratrol, and anthranoids, such as emodin. Emodin and polydatin serve as quality indicators according to the Chinese Pharmacopoeia, with respective minimum contents of 0.6% and 0.15% [5]. According to the European Pharmacopoeia, emodin content of 1.0% and polydatin content of 1.5% are mandatory for quantitative quality assurance of dried plant material [7]. Furthermore, naphthalene derivates such as torachrysone, gallates, catechins and proanthocyanidins are reported [9,10].

*R. japonica* rhizome is a major source of resveratrol for the dietary supplement industry [11]. Resveratrol is used for its antioxidative properties. It can support the therapy of liver ailments following oxidative stress or improve skin condition in combination with oligomeric proanthocyanidins [12,13]. Other stilbenes found in *R. japonica* rhizomes, polydatin and piceatannol also show high antioxidative potential and cytotoxic effects against various cancer cells [14,15]. Resveratrol also exhibits antimicrobial effects against various bacteria, including *Mycobacterium smegmatis*, *Helicobacter pylori*, *Vibrio cholerae*, *Neisseria gonorrhoeae*, *Campylobacter coli* and *Arcobacter cryaerophilus* [16]. For piceatannol, effects against *Staphylococcus aureus* and *Pseudomonas aeruginosa* are reported [17]. The anthranoid emodin is a known stimulant laxative used in treatment of constipation. It also shows anticancer, antibacterial, diuretic and vasorelaxant effects [18]. However, in order to estimate the biological activities of glycosidic plant constituents, the impact of gut microbiota has to be taken into account. The interindividual differences in gut microbiota composition, which is also influenced by individual physiological or pathophysiological conditions, can lead to differences in absorption rates of glycosides as well as their metabolization [19]. In this study, we asked the question of whether traditional preparations like infusion, decoction, and maceration with different ethanol concentrations are suitable methods for extracting the main constituent groups of stilbenes and anthranoids in favorable composition from fresh *R. japonica* rhizome material. We hypothesise that different extraction methods, especially variations in ethanol concentration of macerates, have major influences on yield and composition of extracts and on their antimycobacterial activities. Extracts were evaluated in detail by UHPLC-DAD-ESI-MS^n^ for qualitative, and UHPLC-DAD for quantitative analysis, in validated methods. Since *R. japonica* rhizome extracts and its major constituents showed antimicrobial potential in the past, including good results for pure resveratrol against *M. smegmatis* achieved in our own working group [20], we also evaluated the antimycobacterial effect of raw *R. japonica* extracts for the first time, by investigating their minimum inhibitory concentration (MIC) against *Mycobacterium smegmatis* mc² 155.

## 2. Results

### 2.1. Drug Extract Ratio (DER)

Drug extract ratios were determined for all investigated *R. japonica* rhizome extracts after freeze-drying. Subsequently used abbreviations are as follows: RI for infusion, RD for decoction, and RM for macerates of different ethanol concentrations. Results for DERs are given in Table 1.

Within macerates, yields rose with higher alcohol content of extraction solvent (*p* < 0.01). The extract yield of 12.13% for RD was comparable to the highest macerate yield in RM96 (12.07%) (*p* > 0.05). RM70 and RM38 were similar in extraction efficiency (*p* > 0.05), and both yielded over 10%, while RI gave, by far, the lowest yield of all investigated extraction methods at 3.88% (*p* < 0.01).

### 2.2. Qualitative Analysis via UHPLC-DAD-ESI-MS^n^

Interpretation of DAD-UV and MS^n^ data in negative ionization mode led to the characterization of thirty-four substances in *R. japonica* rhizome extracts by comparison with literature data and available reference compounds. UV spectra of *cis*-resveratrol and *cis*-polydatin obtained by photoisomerization of reference substances aided identification. With the exception of substance **3** (procyanidin dimer Type B), which only seemed to be present in RD, traces of all substances could be found in every extract, confirmed by spectral data and matching retention times (RT). Results are given in Table 2, with threshold for listed fragment ions in MS^n^ set as ≥10% relative intensity.

**Figure 1:**
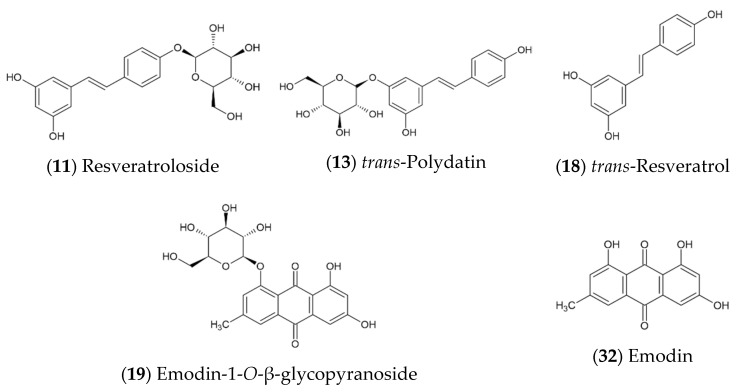
Molecular structures of several identified compounds in *Reynoutria japonica* rhizome extracts belonging to major constituent groups of stilbenes (**11**, **13**, **18**) and anthranoids (**19**, **32**).

Of thirty-four characterized substances, six stilbenes, twelve anthranoids, six proanthocyanidins, epicatechin, catechin, one catechin/epicatechin gallate derivative, two torachrysones, and hydropiperoside were tentatively identified or confirmed by reference substances. Two compounds could be attributed to the class of anthranoids without further identification, another two substances remained undefined. Structural examples, which represent the two main constituent groups of stilbenes and anthranoids, are given in Figure 1.

Concerning substances which could not be directly confirmed with available reference substances and where previously reported findings in literature merely showed derivatives, some additional explanations are needed and presented hereafter. For **16**, −162 amu (m/z 393 to 231) in MS^2^ was indicative of a loss of one hexose unit, leaving demethylated torachrysone. In **25**, the loss of 80 amu in MS^2^ could be attributed to -SO_3_ from a sulfonyl moiety to give torachrysone (m/z 325 to 245). Substance **17** showed −162 amu (m/z 445 to 283) in MS^2^ and **28** a loss of 204 amu in MS^3^ (m/z 487 to 283), which could be attributed to hexose and acetylhexose respectively, both resulting in an aglycone equivalent to emodin methyl ether **30**. In all three substances, this was followed by a loss of 15 amu indicative of -CH_3_ stemming from a methoxy group (m/z 283 to 268), and consequently showing deprotonated fragment ions of emodin **32**. MS^2^ of substance **29** showed loss of CO_2_ at −44 amu (m/z 355 to 311) to give a fragment equivalent to acetylemodin **31**, followed again by deprotonated fragment ions of emodin.

### 2.3. Quantitative Analysis of Stilbenes and Anthranoids via UHPLC-DAD

Quantification was performed for main *trans*-stilbenes and anthranoids in different extracts by UHPLC-DAD. Arithmetic means of triplicate measurements of peak areas (AUC) in UHPLC-DAD chromatograms (λ = 318 nm for stilbenes and 424 nm for anthranoids) were used for calculation. Relative standard deviations (RSD%) for AUCs were <0.60% for stilbenes and < 1.66% for anthranoids, with few exceptions (**2** in RM96, RSD = 4.25%; **6** in RM96, RSD = 2.42%; **6** in RD, RSD = 5.63%). Quantities in freeze-dried extracts, native fluid extracts, and extraction from plant material were calculated from results for sample concentration 2 mg/mL freeze-dried extract in UHPLC analysis, using exact masses of plant material, solvents, and freeze-dried extracts (Supplementary Materials, Table S1). Correction factors (Tables S2 and S3) were applied according to differences in molecular weight of analytes and reference substances. Purity of references was considered and determined to be 99.76% for *trans*-resveratrol and 97.91% for emodin. Results for stilbenes are given as mg/g dry extract after freeze-drying (Table 3), mg/100 g extract solution after filtration (Table 4), and mg/g plant material used for extraction (Table 5), in the same order, results for anthranoids are presented in Table 6, Table 7 and Table 8. Glycoside and aglycone contents represent proportions of total amounts.

**Figure 2:**
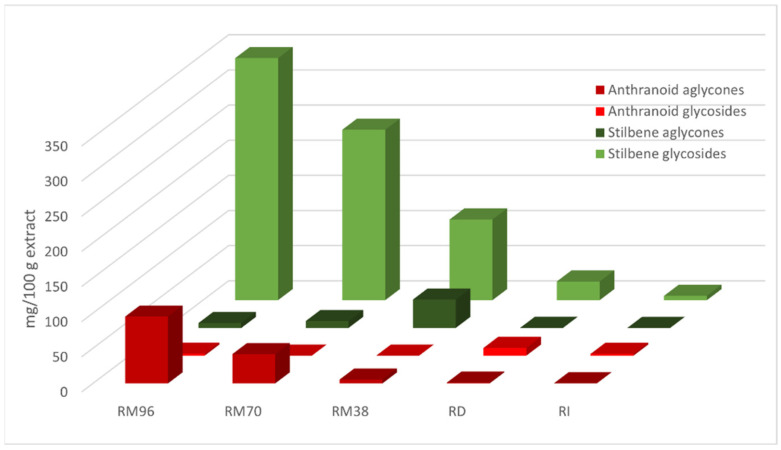
Stilbene and anthranoid glycoside and aglycone content given as mg/100 g fluid extract.

Results for mg/100 g fluid extract (Table 4 and Table 7) showed main stilbenes to be **11** and **13** in all extracts. A comparably high amount of **18** could be found in RM38. The main anthranoid was **32** in all macerates, whereas **23** was dominant in RD and RI. Total stilbene and anthranoid extraction followed the trend RM96 > RM70 > RM38 > RD > RI (*p* < 0.01 for total stilbene and *p* < 0.037 for total anthranoid amount), as did stilbene glycoside (*p* < 0.01) and anthranoid aglycone (*p* < 0.01) extraction (Figure 2). An opposite trend was true for stilbene aglycones in macerates (*p* < 0.01), with highest extraction in RM38, while water extractions still gave the lowest results (*p* < 0.01), with no quantifiable stilbene aglycones in RI. Highest amounts of anthranoid glycosides were extracted in RD (*p* < 0.01), none could be quantified in RM70 and RM38.

**Figure 3:**
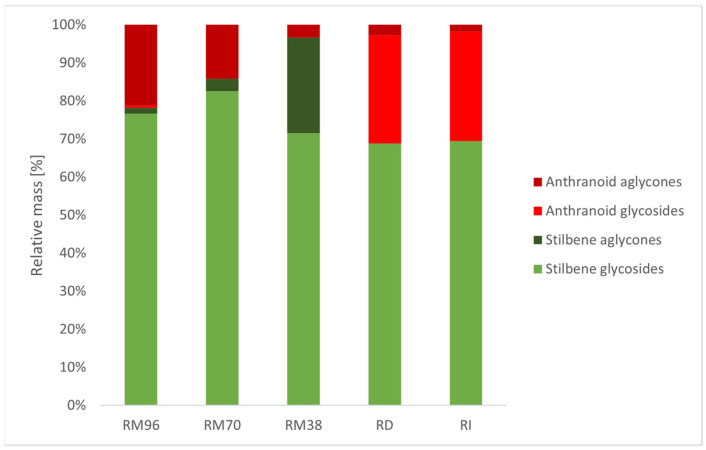
Relative distribution of stilbene and anthranoid glycoside and aglycone content in fluid extracts.

When analysing relative composition of extracts regarding stilbenes and anthranoids, as illustrated in Figure 3, without considering absolute yield, it showed that RM38 had lowest anthranoid content at 3.38% (*p* < 0.01) and a remarkably high fraction of aglycones within stilbenes (25.13%) (*p* < 0.01). RD and RI showed highest anthranoid (31.11 and 30.56%) and total glycoside content (97.13% and 98.20%) in similar relative compositions (Figure 3) (*p* < 0.01 compared to other extraction methods).

#### Validation of Quantitative UHPLC-DAD Method

Linearity of measurements was confirmed for the quantitative UHPLC method used in this study. For reference substance, resveratrol in a working range of 2–300 µg/mL and emodin of 2–500 µg/mL separate correlation coefficients showed (*R^2^*) = 1. Intra-day and inter-day precision were investigated for sample RM96. Results are shown in Table 9. Accuracies were found to be 72.44%, 94.99%, and 94.58% for emodin (3, 200, 400 µg/mL) and 82.85%, 93.64%, and 93.83% for resveratrol (3, 100, 200 µg/mL). For emodin, recovery rates at 3, 200, and 400 µg/mL spike concentrations were determined to be 92.54%, 98.76%, and 98.92%, respectively. For resveratrol at 3, 100, and 200 µg/mL, recovery rates were 109.62%, 96.65%, and 96.81%.

### 2.4. Antimycobacterial Testing of Freeze-Dried Extracts and Reference Substances

MIC testing of freeze-dried extracts showed antimycobacterial effects against *M. smegmatis* for macerates and reference substance resveratrol in the tested concentration ranges (extracts 512–1 µg/mL, reference substances 128–0.25 µg/mL). Infusion and decoction, as well as polydatin and emodin, had no relevant antibacterial effect. Results are summarized in Table 10.

## 3. Discussion

In this study, we analysed different extraction methods of fresh *R. japonica* rhizome, which are easily reproducible without specialized instrumental equipment and using food-grade solvents, to compare yields and composition, particularly focusing on main secondary metabolite groups of anthranoids and stilbenes. UHPLC-DAD-ESI-MS^n^ lead to the characterization of thirty-four substances, of which six were stilbenes, and twelve anthranoids. Substances **16**, **25**, **28,** and **29** (Table 2) can be regarded as new derivatives of previously reported compounds. The main constituents, four *trans*-stilbenes and eight anthranoids, were quantified by means of UHPLC-DAD. Furthermore, we investigated the antimycobacterial potential of extracts and reference compounds against *Mycobacterium smegmatis* in micro-dilution minimum inhibitory concentration (MIC) assays.

Macerates prepared with 38%, 70%, and 96% (*v*/*v*) ethanol (RM38, RM70, RM96) showed yields of 10.32, 10.54, and 12.07% (Table 1), giving the highest ethanol concentration in solvents a slight advantage in yield; however, these did not mark drastic changes in extraction efficiency. Infusion (RI) yields were low at 3.88%, which is to be expected from a short extraction time of 10 min and makes this preparation least suitable for further consideration of usability. Decoction (RD) yielded 12.13% and needs merely 30 min of extraction time compared to three weeks for macerates. However, when extract composition is considered, RD showed the highest anthranoid extraction of all extracts at 31% of quantified substances (Figure 3), which could be considered an undesirable trait of *R. japonica* extracts. Although the European Pharmacopoeia demands minimum contents for polydatin (stilbene) and emodin (anthranoid) in dried *R. japonica* rhizome [7], higher stilbene content and low anthranoid content in extracts seems more desirable to emphasize the antioxidative and antibacterial effects of stilbenes [14,15,16,17], while minimizing the laxative effects of emodin and derivatives [18]. In accordance with the reported results for lyophilized and pulverized *R. japonica* rhizome samples [24], the extraction of anthranoids from fresh and cut *R. japonica* rhizome is rising with ethanol concentration. However, a reported decline in extraction efficiency over 80% ethanol concentration was not found to be occurring in fresh rhizome macerates. From this point of view, RM38 seems most promising, with high stilbene and a remarkably low anthranoid content of 3.38% of quantified substances (5.420 mg/100 g fluid extract, Figure 3, Table 7). Absolute stilbene content amounted to 155.078 mg/100 g fluid extract (Table 4) and could easily be improved through scale-up processes like large batch processing and concentration of constituents by evaporation of solvent. A stilbene extraction from fresh *R. japonica* rhizome with liquified dimethyl ether was reported to yield 0.342 mg/g resveratrol and 2.57 mg/g polydatin, calculated per gram of dry rhizome [25]. Extraction of resveratrol from dried and pulverized *R. japonica* rhizome with deep eutectic solvents (DES) yielded up to 9.00 mg/g dry rhizome [26]. In comparison with both methods, we found RM38 to also yield good quantities of 2.057 mg/g resveratrol and 3.013 mg/g polydatin. Maceration with 38% (*v*/*v*) ethanol can, similarly to DES, be employed without the need to dry and grind plant material, but requires neither specialized equipment, nor complex or critical organic solvents.

The highest relative glycoside content among quantified substances was found in hot water extracts RD and RI (97.13% and 98.20%, Figure 3), which could be due to denaturation of glycosidases by hot water, and therefore prohibition of enzymatic hydrolysis. In macerates, high water content encourages hydrolysis and RM38 showed the lowest glycoside content accordingly (71.49%), entirely made up of stilbene glycosides, with no quantifiable anthranoid glycosides. Interestingly, RM70, and not RM96, showed the highest relative glycoside content among macerates. This could be explained by improved glycoside extraction at 30% (*v*/*v*) water content in solvent, while high ethanol of 70% (*v*/*v*) suffices to stabilize glycosides in solution over longer extraction times.

Antimycobacterial testing against *M. smegmatis* showed good results for all freeze-dried macerates at 256 µg/mL, with no visible effects of different extract compositions regarding stilbenes and anthranoids (Table 10). Water extracts RD and RI, however, showed no significant antibacterial effect (>512 µg/mL), which could be attributed to high glycoside content, and therefore less uptake by bacteria due to higher polarity of compounds. Reference compounds *trans*-resveratrol and *trans*-polydatin support this assumption for stilbenes, with promising results for the aglycone *trans*-resveratrol (64 µg/mL), while glycoside the *trans*-polydatin showed no relevant effect (>128 µg/mL). Anthranoid emodin also showed no inhibitory effect (>128 µg/mL).

As *R. japonica* is highly invasive outside its native regions of Eastern Asia and an aggressive competitor in foreign ecosystems, new perspectives on medicinal use in the West show benefits that might encourage consideration of wild harvest over classic means of eradication, which mostly involve heavy use of herbicides [27]. Extraction and enrichment of stilbenes from fresh plant material can be accomplished by simple extraction methods with food-grade solvents which are also suitable for scale-up, most significantly by maceration with 38% (*v*/*v*) ethanol. Stilbene aglycones of natural origin and their derivatives promise to be interesting targets for further antimycobacterial evaluation.

## 4. Materials and Methods

### 4.1. Plant Material

Rhizomes of *R. japonica* were harvested in September of 2020 in Graz, Austria (47°04′39.38″ N, 15°27′08.90″ E), and stored at +4 °C. A voucher was deposited at the herbarium of the Department of Pharmacognosy, Institute of Pharmaceutical Sciences, University of Graz (specimen voucher: Alperth 010). The plant material was processed the day after collection by washing with cold water and cutting into approximately 2 mm thick slices shortly before extraction. Only pieces with complete cross-sections were used to facilitate comparability between different extraction methods.

### 4.2. Solvents and Reference Substances

Reference substances for qualitative and quantitative analysis were trans-resveratrol (99% purity, Sigma-Aldrich, St. Louis, MO, USA), emodin (90% purity, Sigma-Aldrich), and polydatin CRS (trans-polydatin, 99.1% purity, EDQM—Council of Europe, Strasbourg, France). For extract preparation, deionized water and ethanol (AustrAlco, Spillern, Austria) were used. Samples of reference substances were prepared in p.a. grade methanol (VWR, Radnor, PA, USA). HPLC mobile phases were made up of ultrapure water (Barnstead Easypure RF, Barnstead) and HPLC-grade acetonitrile (VWR).

### 4.3. Photoisomerization of Reference Stilbenes by UV-Exposure

Separate reference solutions of *trans*-resveratrol and *trans*-polydatin were prepared as 1 mg/mL in methanol and treated with UV light (λ = 365 nm) in clear glass vials for 18.5 h to trigger *cis*/*trans*-isomerization for further reference data.

### 4.4. Extract Preparation

Separate samples of fresh plant material were extracted by infusion, decoction, and maceration before filtration with suction and freeze-drying using a VirTis Sentry freeze-dryer (SP Scientific, Warminster, PA, USA). For infusion, 100 g of boiling, deionized water were added to 2 g of cut rhizome in a beaker and covered for an extraction time of 10 min. For decoction, 100 g of cold, deionized water and 2 g of plant material were brought to a boil and then extracted for 30 min under reflux. Macerates were prepared with 20 g of rhizome and 100 g of 38%, 70% and 96% (*v*/*v*) partly denatured ethanol respectively, with an extraction time of three weeks in the dark.

### 4.5. UHPLC-DAD-ESI-MS^n^ for Qualitative Analysis

A Dionex UltiMate 3000 RS Ultra-high performance liquid chromatography (UHPLC) system (Thermo Fisher Scientific, Waltham, MA, USA) comprising pump, autosampler, column compartment and diode array detector (DAD) was used for qualitative analysis of extracts. A Kinetex C18 100 mm × 2.1 mm, 2.6µm column (Phenomenex, Torrance, CA, USA) offered the stationary phase, while the mobile phase consisted of water + 0.1% formic acid (A) and acetonitrile + 0.1% formic acid (B). Runs started at 3% B, increasing to 15% B at 12 min, 42% B at 20 min and 70% B at 23 min, then plateaued until 25 min before dropping back to 3% B at 25.2 min, which was held until 30 min total runtime. Column temperature was kept at 40 °C with flow rate being 0.350 mL/min. DAD recorded spectra in a wavelength range of 190 to 700 nm. For mass spectrometric (MS) detection, the system was coupled to an LTQ XL linear ion-trap mass spectrometer with electrospray ionization (ESI) ion source (Thermo Scientific). Conditions were set to source heater temperature 300 °C, sheath gas flow 40 arb (arbitrary units), auxiliary gas flow 10 arb, source voltage 3.5 kV (ESI neg), and recorded mass range of m/z 50 to 2000 amu.

### 4.6. UHPLC-DAD for Quantitative Analysis

Quantitative analysis was conducted on a separate Dionex UltiMate 3000 RS UHPLC system (Thermo Fisher Scientific), comprising the same modules as for qualitative analysis without coupling to MS. All chromatographic parameters were identical, with extracted quantification wavelengths from DAD-UV detection being 318 nm for stilbenes and 424 nm for anthranoids. Freeze-dried extracts were redissolved in concentrations of 2 mg/mL, with water as solvent for dry extracts from infusion and decoction, and 50% (*v*/*v*) ethanol for macerates. Reference substances were used in 1 mg/mL methanolic stock solutions and subsequent serial dilutions (1:10, 1:100, 1:1000). Injection volumes were 1 and 5 µL for samples and 1 to 5 µL for references according to desired concentration. All measurements were performed in triplicate and quantified substances calculated for their AUC arithmetic means.

### 4.7. Validation of UHPLC-DAD Method

#### 4.7.1. Linearity

To establish linearity of measurements in a working range of 2–300 µg/mL for reference substance, resveratrol concentrations 2, 5, 10, 30, 100, 300 µg/mL were evaluated. For emodin in a working range of 2–500 µg/mL, concentrations 2, 5, 10, 30, 100, 300, 500 µg/mL were used.

#### 4.7.2. Precision

Intra-day and inter-day precision were determined for sample RM96 at a sample concentration of 2 mg/mL in 50% (*v*/*v*) ethanol and injection volume of 1 µL. For intra-day precision, the sample solution was injected six consecutive times in one day and relative standard deviation (RSD) in peak areas (area under the curve, AUC) calculated for four stilbenes and three anthranoids. For inter-day precision, the same sample was injected 3 times each on two consecutive days.

#### 4.7.3. Accuracy

For determination of accuracy, serial dilutions of emodin and resveratrol reference stock solutions were measured in different injection volumes to give target concentrations of 3, 200, and 400 µg/mL for emodin and 3, 100, and 200 µg/mL for resveratrol.

#### 4.7.4. Recovery Rate

Recovery rates for emodin and resveratrol were measured by spiking samples of RM38 at a concentration of 2 mg/mL with reference concentrations also used for determination of accuracy (3, 200, and 400 µg/mL for emodin and 3, 100, and 200 µg/mL for resveratrol).

### 4.8. Antimycobacterial Assay

All freeze-dried extracts and reference compounds were tested in a well-established serial micro-dilution minimum inhibitory concentration (MIC) assay against the bacterial strain *Mycobacterium smegmatis* mc² 155 (ATCC 700084) [28]. Starting concentrations for dilution were 512 mg/L for extracts and 128 mg/L for reference compounds. In short, samples were dissolved in dimethyl sulfoxide (Roth, Karlsruhe, Germany) before serial dilution with Mueller Hinton Broth medium (Oxoid, Basingstoke, Hampshire, UK) in 96-well plates and incubation for 72 hours at 37 °C with a 5 × 10^5^ cfu/mL bacterial inoculum (*n* = 4). Isoniazid (Sigma, St. Louis, MO, USA) served as control antibiotic. Growth and sterile control were included per plate. Evaluation was performed via Methylthiazolyldiphenyl-tetrazoliumbromide (MTT) (Sigma) colorimetric detection, after additional incubation of 30 min.

### 4.9. Statistical Analysis

All statistical analyses were performed in IBM SPSS Statistics 26. To test correlations, Spearman Correlation test was employed as correlations might not be linear and extraction methods are transformed to ordinal variables. For mean comparisons, an ANOVA with a Bonferroni-Holm corrected *t*-test as a post hoc test was used. The α-level was set to 0.05.

## 5. Conclusions

*Reynoutria japonica*, as one of the most critical invasive species in Europe and North America, requires constant attention in management. To shed light on benefits beyond eradication can promote the use of invasive species. As hypothesized, differences in simple extraction methods are apparent. Remarkably, a simple macerate with 38% (*v*/*v*) ethanol gives good yields of stilbenes while showing low anthranoid content and could therefore be a target for large-scale processing of *R. japonica* rhizomes. However, concerning their antimycobacterial potential, all ethanolic macerates showed similar results, both aqueous preparations were less active.

Optimal harvest times for high stilbene content need to be investigated in future research. Also, antimycobacterial stilbene aglycones, their isolation, derivatization, and testing, proved to be promising for future investigation.

## Figures and Tables

**Table 1:** Drug extract ratios for investigated *Reynoutria japonica* rhizome extracts.

Extract ^1^	DER	Yield [%]
RI	25.75:1	3.88%
RD	8.24:1	12.13%
RM96	8.29:1	12.07%
RM70	9.49:1	10.54%
RM38	9.69:1	10.32%

^1^ RI = infusion, RD = decoction, RM = macerates prepared with 96, 70, 38% (*v*/*v*) ethanol.

**Table 2:** Qualitative analysis of *Reynoutria japonica* rhizome extracts.

Substance	RT [min]	Molecular Ion [m/z]	MS^n^ [m/z], Relative Intensity (%)	Molecular Weight [g/mol]	Tentative Identification
**1**	5.15	577 [M-H]^−^	MS^2^[577]: 425 (100), 470 (35), 289 (15) MS^3^[425]: 407 (100), 273 (10) MS^4^[407]: 285 (100), 281 (70), 398 (53), 297 (50)	578	Procyanidin dimer Type B [9,10]
**2**	5.35	335 [M+HCOO]^−^ 289 [M-H]^−^	MS^2^[289]: 245 (100), 205 (38), 179 (15) MS^3^[245]: 203 (100), 227 (30), 187 (25), 161 (20) MS^4^[203]: 175 (100), 188 (65), 161(35), 157 (20)	290	Catechin [9,10,21]
**3**	6.15	577 [M-H]^−^	MS^2^[577]: 425 (100), 470 (35), 289 (15) MS^3^[425]: 407 (100), 273 (10) MS^4^[407]: 285 (100), 281 (70), 398 (53), 297 (50)	578	Procyanidin dimer Type B [9,10]
**4**	6.81	577 [M-H]^−^	MS^2^[577]: 425 (100), 470 (35), 289 (15) MS^3^[425]: 407 (100), 273 (10) MS^4^[407]: 285 (100), 281 (70), 398 (53), 297 (50)	578	Procyanidin dimer Type B [9,10]
**5**	7.46	577 [M-H]^−^	MS^2^[577]: 425 (100), 470 (35), 289 (15) MS^3^[425]: 407 (100), 273 (10) MS^4^[407]: 285 (100), 281 (70), 398 (53), 297 (50)	578	Procyanidin dimer Type B [9,10]
**6**	7.72	335 [M+HCOO]^−^ 289 [M-H]^−^	MS^2^[289]: 245 (100), 205 (38), 179 (15) MS^3^[245]: 203 (100), 227 (30), 187 (25), 161 (20) MS^4^[203]: 175 (100), 188 (65), 161(35), 157 (20)	290	Epicatechin [9,10,21]
**7**	7.97	383 [M-H]^−^	MS^2^[383]: 303 (100), 285 (13) MS^3^[303] 285 (100) MS^4^[285]: 241 (100), 175 (50), 243 (20), 199 (18), 257 (15), 217 (15)	384	Undefined
**8**	8.77	186 [M-H]^−^	MS^2^[186]: 125 (100) MS^3^[125]: 97 (100), 125 (35), 57 (18)	187	Undefined
**9**	9.88	865 [M-H]^−^	MS^2^[865]: 695 (100), 577 (68), 739 (65), 575 (35), 425 (25), 847 (22), 287 (20) MS^3^[695]: 543 (100), 451 (38), 677 (35), 405 (32), 243 (30), 525 (28), 289 (17)	866	Procyanidin trimer Type B [10]
**10**	9.90	405 [M-H]^−^	MS^2^[405]: 243 (100) MS^3^[243]: 225 (100), 201 (50), 199 (35), 175 (30) MS^4^[225]: 157 (100), 197 (60), 181 (45), 225 (30)	406	*trans*-Piceatannol-hexoside [9,10,21]
**11**	10.13	435 [M+HCOO]^−^	MS^2^[435]: 289 (100), 227 (13) MS^3^[289]: 227 (100) MS^4^[227]: 185 (100), 183 (50), 157 (40), 159 (40)	390	Resveratroloside [10,21]
**12**	10.40	729 [M-H]^−^	MS^2^[729]: 559 (100), 407 (95), 577 (93), 441 (75), 603 (70), 451 (55), 711 (30), 289 (28)	730	Procyanidin dimer monogallate [10]
**13**	11.86	435 [M+HCOO]^−^	MS^2^[435]: 289 (100) MS^3^: 227 (100) MS^4^[227]: 185 (100), 183 (50), 157 (40), 159 (40)	390	*trans*-Polydatin ^1^
**14**	12.08	441 [M-H]^−^	MS^2^[441]: 289 (100), 169 (22), 331 (18) MS^3^[289]: 245 (100), 205 (38), 179 (18) MS^4^[245]: 203 (100), 127 (25), 187 (22), 161 (20), 217 (10)	442	Catechin/Epicatechin monogallate [9]
**15**	14.86	435 [M+HCOO]^−^	MS^2^[435]: 289 (100) MS^3^: 227 (100) MS^4^[227]: 185 (100), 183 (50), 157 (40), 159 (40)	390	*cis*-Polydatin ^2^
**16**	15.12	393 [M-H]^−^ 505 [M-H]^−^	MS^2^[393]: 231 (100) MS^2^[505]: 289 (100), 215 (38)	394 (1) 506 (2)	Demethylated torachrysone- hexoside [9,10] (1), Undefined (2)
**17**	15.43	445 [M-H]^−^	MS^2^[445]: 283 (100) MS^3^[283]: 240 (100), 265 (70), 268 (35)	446	Emodin methyl ether hexoside [22]
**18**	15.58	227 [M-H]^−^	MS^2^[227]: 185 (100), 183 (58), 157 (50), 159 (47)	228	*trans*-Resveratrol ^1^
**19**	15.97	431 [M-H]^−^	MS^2^[431]: 269 (100), 311 (10) MS^3^[269]: 225 (100), 241 (18) MS^4^[225]: 181 (100), 210 (80), 225 (65), 197 (25)	432	Emodin-1-*O*-β-glucopyranoside [9,10]
**20**	16.25	511 [M-H]^−^	MS^2^[511]: 269 (100), 431 (43) MS^3^[296]: 225 (100), 241 (25), 269 (15)	512	Emodin-*O*-(sulfonyl)-hexoside [9,10]
**21**	16.87	299 [M-H]^−^	MS^2^[299]: 256 (100), 284(25) MS^3^[256]: 227 (100), 228 (70), 226 (65), 239 (26), 211 (20), 256 (20)	300	Undefined anthranoid
**22**	16.9	273 [M+HCOO]^−^	MS^2^[273]: 227(100) MS^3^[227]: 185 (100), 183 (55), 157 (40), 159 (40)	228	*cis*-Resveratrol ^2^
**23**	17.49	431 [M-H]^−^	MS^2^[431]: 269 (100), 311 (10) MS^3^[269]: 225 (100), 241 (18) MS^4^[225]: 181 (100), 210 (83), 225 (63), 197 (25)	432	Emodin-hexoside [9,10]
**24**	18.20	517 [M-H]^−^ 1035 [2M-H]^−^	MS^2^[517]: 473 (100) MS^3^[473]: 269 (100), 311 (10), MS^4^[269]: 225 (100), 241 (18)	518	Emodin-8-*O*- (6′-*O*-malonyl)- hexoside [9,10]
**25**	18.46	325 [M-H]^−^	MS^2^[325]: 245 (100) MS^3^[245]: 230 (100) MS^4^[245]: 215 (100)	326	Sulfonyl- torachryson [9]
**26**	18.7	285 [M-H]^−^	MS^2^[285]: 241 (100), 257 (45), 285 (30), 211 (10) MS^3^[241]: 211 (100), 195 (52), 212 (35), 224 (35)	286	Hydroxyemodin [23]
**27**	18.87	779 [M-H]^−^	MS^2^[779]: 633 (100), 615 (12), 487 (10) MS^3^[633]: 487 (100), 453 (25), 469 (20)	780	Hydropiperoside [10]
**28**	19.57	533 [M+HCOO]^−^	MS^2^[533]: 487 (100), 283 (92), 486 (20) MS^3^[487]: 283 (100), 427 (35), 455 (33), 409 (25), 469 (15) MS^3^[283]: 240 (100), 268 (40)	488	Emodin methyl ether acetylhexoside [22]
**29**	19.80	355 [M-H]^−^	MS^2^[355]:311 (100) MS^3^[311]: 267 (100), 268 (65), 283 (35), 311 (15)	356	Malonylemodin [9]
**30**	20.94	283 [M-H]^−^	MS^2^[283]: 240 (100), 268 (30) MS^3^[240]: 212 (100), 240 (33), 196 (10), 184 (10) MS^4^[212]: 184 (100)	284	Emodin methyl Ether [22]
**31**	22.38	311 [M-H]^−^	MS^2^[311]: 267 (100), 268 (50), 283 (60), 311 (43) MS^3^[267]: 224 (100), 225 (33), 240 (30), 223 (12) MS^4^[224]: 196 (100), 195 (15)	312	Acetylemodin [9]
**32**	22.79	269 [M−H]^−^	MS^2^[269]: 225 (100), 241 (24), 269 (15) MS^3^[225]: 281 (100), 210 (73), 225 (63), 197 (30) MS^4^[281]: 181 (100), 153 (10)	270	Emodin ^1^
**33**	23.81	509 [M-H]^−^	MS^2^[509]: 254 (100), 491(10) MS^3^[254]: 226 (100), 254 (23), 225 (15)	510	Emodin-bianthron [10]
**34**	24.51	353 [M-H]^−^	MS^2^[353]: 123 (100), 335 (15), 309 (12), MS^3^[123]: 123 (100), 95 (65)	354	Undefined anthranoid

^1^ identified by comparison with reference compound; ^2^ identified after UV-isomerization of reference compound.

**Table 3:** Quantitative analysis of stilbenes in *Reynoutria japonica* rhizome extracts calculated as mg/g freeze-dried extract.

Extract	10	11	13	18	Total	Glycosides	Aglycones
RI	2.669	29.975	47.883	- ^1^	80.527	80.527	-
RD	3.618	40.132	67.427	0.236	111.413	111.177	0.236
RM96	4.968	53.561	87.993	2.952	149.474	146.521	2.952
RM70	4.014	42.334	71.068	4.684	122.100	117.416	4.684
RM38	2.024	25.473	29.192	19.926	76.615	56.689	19.926

^1^ = not quantifiable.

**Table 4:** Quantitative analysis of stilbenes in *Reynoutria japonica* rhizome extracts calculated as mg/100 g fluid extract.

Extract	10	11	13	18	Total	Glycosides	Aglycones
RI	0.205	2.302	3.678	- ^1^	6.185	6.185	-
RD	0.865	9.590	16.112	0.057	26.623	26.567	0.057
RM96	11.676	125.880	206.802	6.938	351.296	344.358	6.938
RM70	8.293	87.464	146.829	9.678	252.264	242.587	9.678
RM38	4.096	51.561	59.087	40.333	155.078	114.745	40.333

^1^ = not quantifiable.

**Table 5:** Quantitative analysis of stilbenes in *Reynoutria japonica* rhizome extracts calculated as mg/g plant material.

Extract	10	11	13	18	Total	Glycosides	Aglycones
RI	0.104	1.164	1.860	- ^1^	3.128	3.128	-
RD	0.439	4.868	8.179	0.029	13.515	13.487	0.029
RM96	0.600	6.464	10.619	0.356	18.039	17.683	0.356
RM70	0.423	4.460	7.488	0.494	12.865	12.371	0.494
RM38	0.209	2.629	3.013	2.057	7.907	5.851	2.057

^1^ = not quantifiable.

**Table 6:** Quantitative analysis of anthranoids in *Reynoutria japonica* rhizome extracts calculated as mg/g freeze-dried extract.

Extract	19	23	24	26	28	30	32	34	Total	Glycosides	Aglycones
RI	2.459	24.433	3.540	0.996	2.919	0.000	1.087	- ^1^	35.433	33.350	2.084
RD	4.002	35.318	3.663	2.461	2.923	0.221	1.717	-	50.307	45.907	4.400
RM96	-	1.218	-	-	-	3.104	35.422	1.920	41.664	1.218	40.446
RM70	-	-	-	-	-	2.184	17.170	0.791	20.145	-	20.145
RM38	-	-	-	-	-	1.172	1.505	-	2.678	-	2.678

^1^ = not quantifiable.

**Table 7:** Quantitative analysis of anthranoids in *Reynoutria japonica* rhizome extracts calculated as mg/100 g fluid extract.

Extract	19	23	24	26	28	30	32	34	Total	Glycosides	Aglycones
RI	0.189	1.877	0.272	0.077	0.224	- ^1^	0.083	-	2.721	2.561	0.160
RD	0.956	8.440	0.875	0.588	0.698	0.053	0.410	-	12.021	10.970	1.051
RM96	-	2.863	-	-	-	7.294	83.250	4.512	97.919	2.863	95.056
RM70	-	-	-	-	-	4.511	35.475	1.634	41.620	-	41.620
RM38	-	-	-	-	-	2.373	3.047	-	5.420	-	5.420

^1^ = not quantifiable.

**Table 8:** Quantitative analysis of anthranoids in *Reynoutria japonica* rhizome extracts calculated as mg/g plant material.

Extract	19	23	24	26	28	30	32	34	Total	Glycosides	Aglycones
RI	0.095	0.949	0.137	0.039	0.113	- ^1^	0.042	-	1.376	1.295	0.081
RD	0.485	4.284	0.444	0.299	0.355	0.027	0.208	-	6.103	5.569	0.534
RM96	-	0.147	-	-	-	0.375	4.275	0.232	5.028	0.147	4.881
RM70	-	-	-	-	-	0.230	1.809	0.083	2.123	-	2.123
RM38	-	-	-	-	-	0.121	0.155	-	0.276	-	0.276

^1^ = not quantifiable.

**Table 9:** Intra-day and inter-day precision RSD (%) for several quantified stilbenes (**10**–**18**) and anthranoids (**30**–**34**).

Substance	Intra-Day Precision RSD [%]	Inter-Day Precision RSD [%]
**10**	0.3612	0.9405
**11**	0.3318	0.8861
**13**	0.4201	1.0069
**18**	0.6999	1.1285
**30**	3.8111	2.6544
**32**	0.7511	1.5208
**34**	6.2563	7.7805

**Table 10:** Minimum inhibitory concentrations of freeze-dried extracts on *Mycobacterium smegmatis* mc^2^ 155 growth (*n* = 4).

Sample	MIC [µg/mL]
RI	>512
RD	>512
RM96	256
RM70	256
RM38	256
*trans*-Resveratrol	64
*trans*-Polydatin	>128
Emodin	>128

## Data Availability

Data are included in this article.

## Supplementary Materials

The following are available online at https://www.mdpi.com/article/10.3390/plants10091809/s1, Table S1: Data used for quantitative calculations, Table S2: Correction factors for stilbene quantification relative to reference *trans*-resveratrol, Table S3: Correction factors for anthranoid quantification relative to reference emodin.

## References

[1] WFO *Reynoutria japonica* Houtt. http://www.worldfloraonline.org/taxon/wfo-0000406106.

[2] Bailey J.P., Conolly A.P. (2000). Prize-Winners to Pariahs—A History of Japanese Knotweed s.l. (*Polygonaceae*) in the British Isles. Watsonia.

[3] Jones D., Bruce G., Fowler M.S., Law-Cooper R., Graham I., Abel A., Street-Perrott F.A., Eastwood D. (2018). Optimising physiochemical control of invasive Japanese knotweed. Biol. Invasions.

[4] Global Invasive Species Database Species Profile: *Polygonum cuspidatum*. http://www.iucngisd.org/gisd/speciesname/Polygonum+cuspidatum.

[5] Peng W., Qin R., Li X., Zhou H. (2013). Botany, phytochemistry, pharmacology, and potential application of *Polygonum cuspidatum* Sieb.et Zucc.: A review. J. Ethnopharmacol..

[6] Zhang H., Li C., Kwok S.-T., Zhang Q.-W., Chan S.-W. (2013). A Review of the Pharmacological Effects of the Dried Root of *Polygonum cuspidatum* (Hu Zhang) and Its Constituents. Evid. Based Complement. Altern. Med..

[7] (2020). Europäisches Arzneibuch: 10. Ausgabe, Grundwerk. Polygoni Cuspidati Rhizoma et Radix.

[8] Nawrot-Hadzik I., Hadzik J., Fleischer M., Choromańska A., Sterczała B., Kubasiewicz-Ross P., Saczko J., Gałczyńska-Rusin M., Gedrange T., Matkowski A. (2019). Chemical Composition of East Asian Invasive Knotweeds, their Cytotoxicity and Antimicrobial Efficacy Against Cariogenic Pathogens: An In-Vitro Study. Med. Sci. Monit..

[9] Fu J., Wang M., Guo H., Tian Y., Zhang Z., Song R. (2015). Profiling of components of rhizoma et radix polygoni cuspidati by high-performance liquid chromatography with ultraviolet diode-array detector and ion trap/time-of-flight mass spectrometric detection. Pharmacogn. Mag..

[10] Nawrot-Hadzik I., Ślusarczyk S., Granica S., Hadzik J., Matkowski A. (2019). Phytochemical Diversity in Rhizomes of Three Reynoutria Species and their Antioxidant Activity Correlations Elucidated by LC-ESI-MS/MS Analysis. Molecules.

[11] Chen H., Tuck T., Ji X., Zhou X., Kelly G., Cuerrier A., Zhang J. (2013). Quality assessment of Japanese knotweed (*Fallopia japonica*) grown on Prince Edward Island as a source of resveratrol. J. Agric. Food Chem..

[12] Ahmad A., Ahmad R. (2014). Resveratrol mitigate structural changes and hepatic stellate cell activation in N′-nitrosodimethylamine-induced liver fibrosis via restraining oxidative damage. Chem. Biol. Interact..

[13] Buonocore D., Lazzeretti A., Tocabens P., Nobile V., Cestone E., Santin G., Bottone M.G., Marzatico F. (2012). Resveratrol-procyanidin blend: Nutraceutical and antiaging efficacy evaluated in a placebocontrolled, double-blind study. Clin. Cosmet. Investig. Dermatol..

[14] Piotrowska H., Kucinska M., Murias M. (2012). Biological activity of piceatannol: Leaving the shadow of resveratrol. Mutat. Res..

[15] Su D., Cheng Y., Liu M., Liu D., Cui H., Zhang B., Zhou S., Yang T., Mei Q. (2013). Comparision of piceid and resveratrol in antioxidation and antiproliferation activities in vitro. PLoS ONE.

[16] Vestergaard M., Ingmer H. (2019). Antibacterial and antifungal properties of resveratrol. Int. J. Antimicrob. Agents.

[17] Mattio L.M., Dallavalle S., Musso L., Filardi R., Franzetti L., Pellegrino L., D’Incecco P., Mora D., Pinto A., Arioli S. (2019). Antimicrobial activity of resveratrol-derived monomers and dimers against foodborne pathogens. Sci. Rep..

[18] Zheng Y.-F., Liu C.-F., Lai W.-F., Xiang Q., Li Z.-F., Wang H., Lin N. (2014). The laxative effect of emodin is attributable to increased aquaporin 3 expression in the colon of mice and HT-29 cells. Fitoterapia.

[19] Braune A., Blaut M. (2016). Bacterial species involved in the conversion of dietary flavonoids in the human gut. Gut Microbes.

[20] Lechner D., Gibbons S., Bucar F. (2008). Plant phenolic compounds as ethidium bromide efflux inhibitors in Mycobacterium smegmatis. J. Antimicrob. Chemother..

[21] Fan P., Hay A.-E., Marston A., Lou H., Hostettmann K. (2009). Chemical variability of the invasive neophytes *Polygonum cuspidatum* Sieb. and Zucc. and *Polygonum sachalinensis* F. Schmidt ex Maxim. Biochem. Syst. Ecol..

[22] Feng J., Ren H., Gou Q., Zhu L., Ji H., Yi T. (2016). Comparative analysis of the major constituents in three related polygonaceous medicinal plants using pressurized liquid extraction and HPLC-ESI/MS. Anal. Methods.

[23] Kimura Y., Kozawa M., Baba K., Hata K. (1983). New Constitutents of Roots of *Polygonum cuspidatum*. Planta Med..

[24] Glavnik V., Vovk I. (2020). Extraction of Anthraquinones from Japanese Knotweed Rhizomes and Their Analyses by High Performance Thin-Layer Chromatography and Mass Spectrometry. Plants.

[25] Kanda H., Oishi K., Machmudah S., Wahyudiono, Goto M. (2021). Ethanol-free extraction of resveratrol and its glycoside from Japanese knotweed rhizome by liquefied dimethyl ether without pretreatments. Asia-Pac. J. Chem. Eng..

[26] Sun B., Zheng Y.-L., Yang S.-K., Zhang J.-R., Cheng X.-Y., Ghiladi R., Ma Z., Wang J., Deng W.-W. (2021). One-pot method based on deep eutectic solvent for extraction and conversion of polydatin to resveratrol from *Polygonum cuspidatum*. Food Chem..

[27] Delbart E., Mahy G., Weickmans B., Henriet F., Crémer S., Pieret N., Vanderhoeven S., Monty A. (2012). Can land managers control Japanese knotweed? Lessons from control tests in Belgium. Environ. Manag..

[28] Stavri M., Schneider R., O’Donnell G., Lechner D., Bucar F., Gibbons S. (2004). The antimycobacterial components of hops (*Humulus lupulus*) and their dereplication. Phytother. Res..

