# Supplementary material for: UHPLC Analysis of Reynoutria japonica Houtt. Rhizome Preparations Regarding Stilbene and Anthranoid Composition and Their Antimycobacterial Activity Evaluation
Source: Plants (Basel). 2021 Aug 30;10(9):1809. doi: 10.3390/plants10091809 (PMC8470514; doi:10.3390/plants10091809)
Supplement: Supplementary file 1 [file plants-10-01809-s001.zip › plants-1347214-supplementary.pdf]

Article

UHPLC Analysis of *Reynoutria japonica* Houtt. Rhizome Preparations Regarding Stilbene and Anthranoid Composition and their Antimycobacterial Activity Evaluation

Fabian Alperth [1], Lena Melinz [1], Johannes-Paul Fladerer [1] and Franz Bucar [1,*]

[1] Institute of Pharmaceutical Sciences, University of Graz, Beethovenstraße 8, 8010 Graz, Austria; fabian.alperth@uni-graz.at; lena.melinz@gmail.com; johannes.fladerer@uni-graz.at
* Correspondence: franz.bucar@uni-graz.at; Tel.: +43-316-380-5531

Supplementary Materials

Extract concentrations in mg freeze-dried extract per g solvent were converted to mg/mL under consideration of different densities of ethanolic solutions. Relation of these concentrations to UHPLC sample concentration of 2 mg freeze-dried extract per mL gave multiplicators, which were used to adjust quantitative results to represent extract compositions accurately. Data including exact masses of plant materials for extract preparations are given in Table S1.

Table S1. Data used for quantitative calculations.

Sample	Plant Material [g]	Freeze-dried Extract Yield [g]	Solvent [g] / [ml]	Extract concentration [mg/ml]	Multiplicator
RM96<br>96% Ethanol<br>ϱ = 0.80742 g/ml	20.0368	2.4181	100.47 / 124.43	19.433	9.7164
RM70<br>70% Ethanol<br>ϱ = 0.88556 g/ml	20.0547	2.113	100.16 / 113.10	18.682	9.3410
RM38<br>ϱ = 0.95118 g/ml	20.0373	2.068	100.10 / 105.24	19.651	9.8254
RI<br>Water	1.9877	0.0772	100.44 / 100.44	0.76862	0.3843
RD<br>Water	1.9809	0.2403	100.32 / 100.32	2.39533	1.1977

Correction factors (Tables S2 and S3) were applied according to differences in molecular weight of analytes and reference substances.

Table S2. Correction factors for stilbene quantification relative to reference *trans*-resveratrol

Stilbenes	M [g/mol]	Factor
trans-Resveratrol (**18**) [1]	228	-
trans-Piceatannol-hexoside (**10**)	406	1.781
Resveratroloside (**11**)	390	1.711
trans-Polydatin (**13**)	390	1.711

[1] Reference standard

Table S3. Correction factors for anthranoid quantification relative to reference emodin

Anthranoids	M [g/mol]	Factor
Emodin (**32**) [1]	270	-
Emodin-1-*O*-β-glycopyranoside (**19**)	432	1.600
Emodin- hexoside (**23**)	432	1.600
Emodin-8-*O*-(6'-*O*-malonyl)-hexoside (**24**)	518	1.919
Hydroxyemodin (**26**)	286	1.059
Emodin methyl ether + acetylhexose (**28**)	488	1.807
Emodin methyl ether (**30**)	284	1.052
Undefined anthranoid (**34**)	354	1.311

[1] Reference standard
